# Supplementary material for: MetaRibo-Seq measures translation in microbiomes
Source: Nat Commun. 2020 Jun 29;11:3268. doi: 10.1038/s41467-020-17081-z (PMC7324362; doi:10.1038/s41467-020-17081-z)
Supplement: Supplementary file 10 — Supplementary Data 7 [file 41467_2020_17081_MOESM10_ESM.zip › File2/Confidence_High_Taxonomy/233934_out.krona.html]

Javascript must be enabled to view this page.

members
magnitude
magnitudeUnassigned
count
unassigned
taxon
rank

233934\_out

3

2
superkingdom
3

3
phylum
1239

class
186801
3

186802
order
3

3
186803
family


SRS1041033\_contig\_number\_contig-100\_772.145858
1
2292903
species

2
207244
genus

2

SRS142503\_contig\_number\_50153SRS149879\_contig\_number\_22120
649756
species
